# Removing hardware from anterior approaches following acetabular fractures: a challenging yet indicated procedure

**DOI:** 10.1007/s00264-024-06383-2

**Published:** 2024-11-27

**Authors:** Ahmed Khalifa, Ali Fergany, Bahaaeldin Ibrahim, Osama Farouk

**Affiliations:** 1 Assiut University Hospitals, Assiut, Egypt; 2 South Valley University, Qina, Egypt; 3 Al Azhar University, Assiut, Egypt

**Keywords:** Anterior approaches, Acetabular fracture, Hardware removal, Modiffied stoppa, Ilioinguinal approach, Pararectus approach

## Abstract

**Purpose:**

To describe the indications, outcomes, and incidence of complications after hardware removal from anterior approaches following acetabular fractures.

**Materials:**

Over ten years, 13 patients were included, complaining of pain due to late infection in nine (69.2%) and secondary osteoarthritis in four (30.8%). Fractures classification were T-type fracture (46.2%), both columns (38.5%), one transverse (7.7%), and one (7.7%) T-type with a posterior wall. The approaches utilized for hardware removal were modified Stoppa in 11 (84.6%) patients, ilioinguinal lateral (iliac) window in nine (69.2%), Pararectus in one (7.7%), ilioinguinal in one (7.7%), and Kocher-Langenbeck approach in one (7.7%)).

**Results:**

The patients’ mean age was 37.1 ± 14.9 (21 to 65) years, and nine (69.2%) were males. Hardware removal was performed after the index surgery by a mean of 35.6 ± 20 months. The mean operative time was 143.8 ± 36 min, and the mean blood loss was 1573 ± 842 CC. The mean hospital stay was 3.2 ± 2.3 days, and all patients required blood transfusion. Four (30.8%) intraoperative complications, two (15.4%) vascular injuries, One (7.7%) urinary bladder injury, and in two (15.4%) broken screws could not be retrieved. Postoperative complications in five (38.5%): three (23.1%) had superficial wound infection, one (7.7%) had DVT, and one (7.7%) had L5 nerve root injury. After a mean follow up of 11.3 ± 4.4 (6 to 20) months, the VAS score decreased from a preoperative median of 6 (2 to 8) to a median score of 1 (0 to 6) at the last follow up. 11 (84.6%) patients described the pain as none or occasional, and eight (61.5%) were very satisfied with the results.

**Conclusion:**

Hardware removal from the anterior approaches after acetabular fractures is demanding and carries a high complication risk. The surgeries should be performed when highly indicated, and the surgical team must be familiar with the anterior approaches.

**Supplementary Information:**

The online version contains supplementary material available at 10.1007/s00264-024-06383-2.

## Introduction

Hardware removal after various upper and lower limb fracture fixation surgeries is controversial; moreover, with the paucity of evidence, it is more challenging when it comes to implant removal after acetabular fractures [1–5].

Although surgeons believe that asymptomatic implant retention is safe [6], in cases presenting with fracture-related infection (FRI), perforating implants, and implants causing an allergic reaction, some surgeons argue that hardware removal is absolutely indicated [2]. While in growing children, hardware causes irritating tenosynovitis and hardware breakage, and, at the patient’s request, it could be considered debatable or have relative indications [2, 3, 7].

Hardware removal is a surgical intervention that carries all the typical risks related to anaesthesia and surgery [2, 8]; with an incidence of complications reaching up to 20% [9]; furthermore, the procedure itself could be frustrating, as described by some surgeons, owing to the need for extended exposure with the possibility of increased blood loss (sometimes it is needed even when the index surgery was performed through minimally invasive approaches of MIPO), the chance of implants breakage and inability to remove all implants, and unresolved symptoms postoperatively leading to patients dissatisfaction [5, 8, 10].

The acetabular fracture hardware removal could be indicated to remove hip joint penetrating implants or as a preparatory step before performing total hip arthroplasty (THA) in cases that developed secondary osteoarthritis (OA) [11, 12]. However, the removal of hardware procedures becomes more complex due to the anatomical nature of this area, the proximity of the major neurovascular bundle, and the possibility of dense adhesions [11, 13–15]. However, the literature on hardware removal after acetabular fractures (particularly those operated upon through anterior-type approaches) is scarce.

So, our objective was to report our early results, challenges, indications, and complications in a group of patients who underwent hardware removal after they initially had anterior fixation of acetabular fractures through various types of anterior approaches.

## Patients and methods

This case series is reported per CARE guidelines (Supplementary file 1). It is a part of patient follow-up (included in a previous study [16]) for those who were treated at our institution (a specialized pelvis and acetabulum trauma unit at a North African level one trauma centre) for acetabular fractures that were treated through anterior approaches. The institutional review board approved the original study (Approval No.: 17200352). Furthermore, all the procedures were performed following Helsinki declarations, and informed consent was obtained from all participants.

### Inclusion and exclusion criteria

Between January 2014 and April 2024, patients who underwent hardware removal and had a complete follow up were included, while who had incomplete follow up, and those who refused to participate in the study were excluded. This led to the final inclusion of 13 patients.

### Preoperative Assessment

**Fig. 1:**
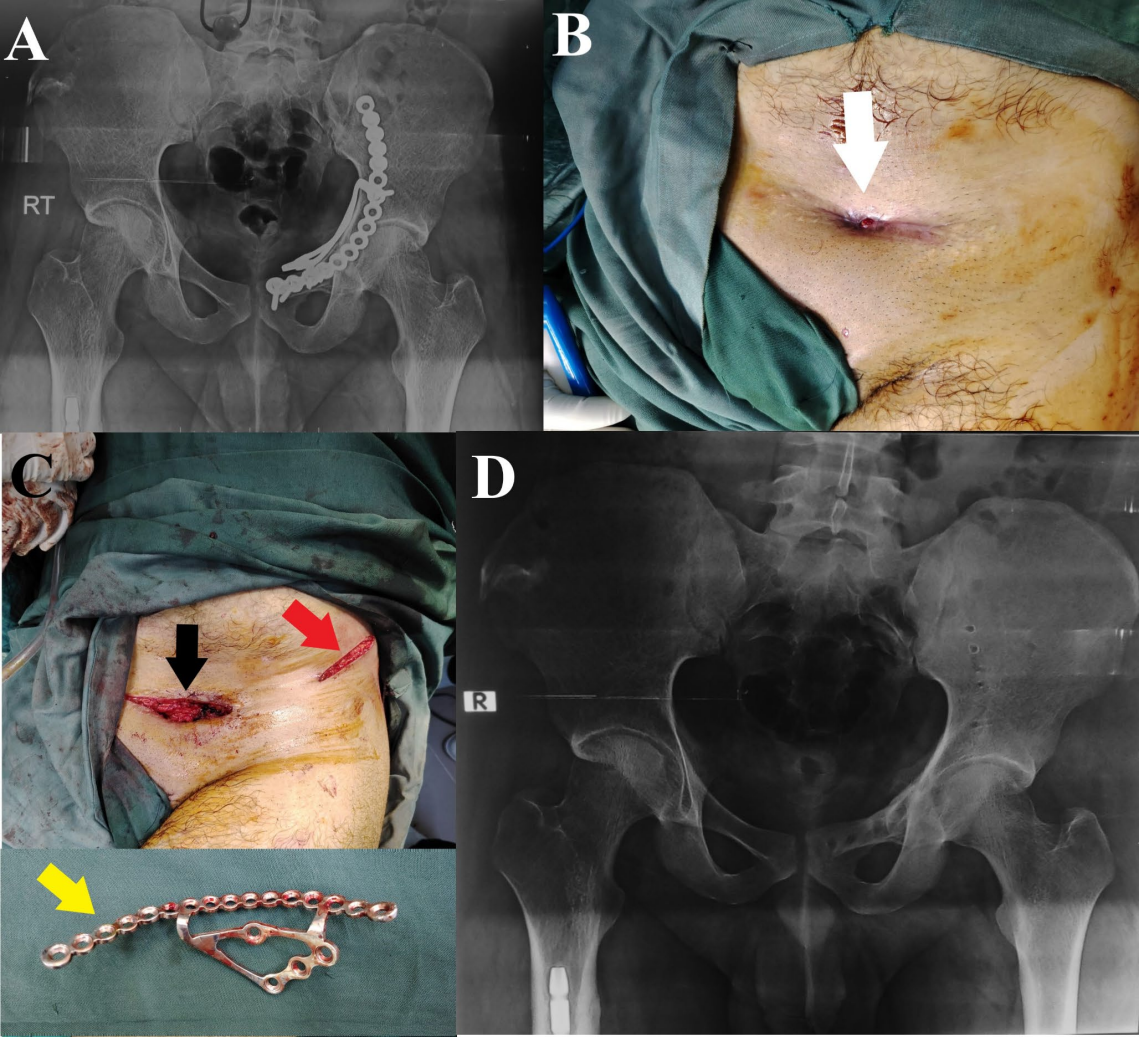
27-year-old male patient who had a T-type fracture treated by ORIF, hardware was performed through a modified Stoppa approach and a lateral window after 24 months of the index surgery. **A**, preoperative. **B**, a clinical image showing the discharging sinus (white arrowhead). **C**, an intraoperative image showing the modified Stoppa approach (black arrowhead), the ilioinguinal approach iliac window (red arrowhead), and the retrieved implant (yellow arrowhead). **D**, postoperative anteroposterior radiograph. (Patient No. 13 in Supplementary File 2)

**Fig. 2:**
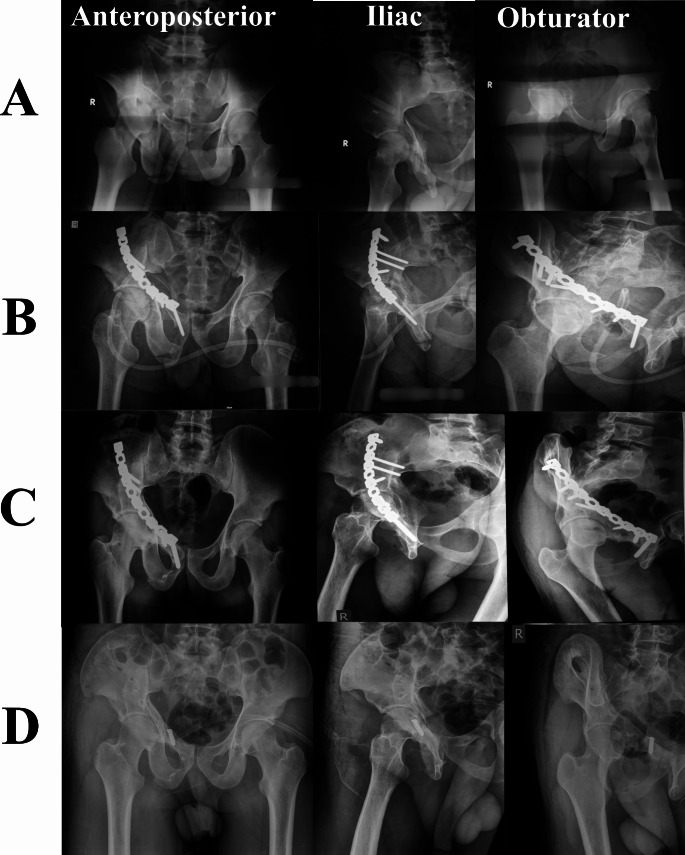
23-year-old male patient who had a T-type fracture treated by ORIF, hardware was performed through a modified Stoppa approach and a lateral window after 30 months of the index surgery. **A**, plain radiographs of the index injury. **B**, follow up after ORIF. **C**, pre-hardware removal. **D**, after hardware removal. (Patient No. 3 in Supplementary File 2)

**Fig. 3:**
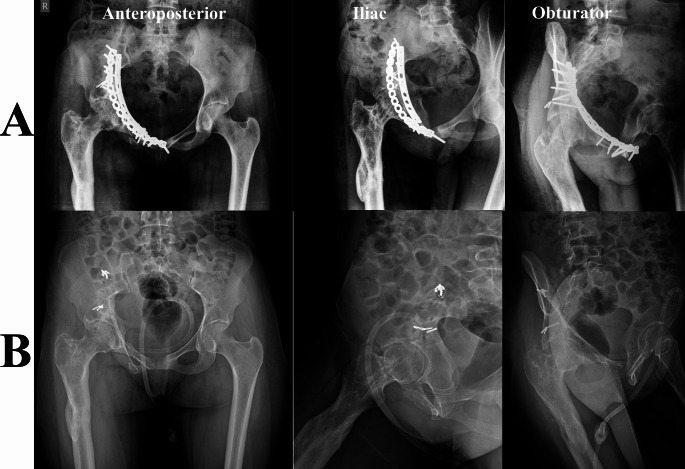
32-year-old female patient who had a T-type fracture treated by ORIF, hardware was performed through a modified Stoppa approach and a lateral window after 63 months of the index surgery. **A**, preoperative plain radiographs. **B**, postoperative. (Patient No. 7 in Supplementary File 2)

*History*: All patients’ initial injuries, index operation details, and postoperative course data were obtained from our Pelvic registry. A detailed history of the presenting symptoms necessitating hardware removal, including pain and its nature and history suggesting peri-implant infection, was obtained. *Clinical evaluation*: the scar of the previous surgery was evaluated (mobility, signs of infection, presence of sinus (Fig. 1)), sciatic nerve status was documented, and the functional assessment of the hip joint. *Imaging studies*: All patients had a plain radiographic anteroposterior (AP) view of the pelvis and Judet oblique views (obturator and iliac) (Figs. 2 and 3). *Laboratory test*: Besides routine preoperative laboratory tests, differential white blood cell (WBC) count, ESR, and CRP were obtained as part of the FRI workup [17, 18].

### Operative Management

After detailed counseling with patients regarding the possible complications of the surgery, we made sure that general, vascular, and urology surgeons were informed about the surgery in case we needed their intervention. Furthermore, the operative room was equipped with fluoroscopy, and a screw removal set was available.

All procedures were performed under general anaesthesia while the patient was in a supine position on a radiolucent table. A Foley catheter was inserted for bladder drainage to ensure its protection. Draping was performed in a usual manner.

### Surgical approaches

The same surgical team involved in the index surgeries was engaged in the hardware removal procedures. We followed the same surgical incision of the index surgeries (The modified Stoppa approach was utilized in 11 (84.6%) patients, the lateral (iliac) window of the ilioinguinal approach was needed in nine (69.2%), the Pararectus approach in one (7.7%), the ilioinguinal approach in one (7.7%), and Kocher-Langenbeck approach in one (7.7%)). Meticulous soft tissue dissection (under vision) was followed in all cases, especially if there was an infection (due to tissue friability). In Modified Stoppa approach, the dissection started from the symphysis pubis; dissection was carried out till reaching the bladder, which was identified, protected, and retracted. Various retractors (such as double-angled Homan retractors) were used to protect the soft tissue structures (neurovascular bundles) throughout the procedure, ensuring that the assistant implemented gentle retraction.

After reaching the site of the plate and screws, careful dissection of the soft tissues around using a periosteal elevator till total exposure of the plate and screws. Soft tissue samples around the plate were obtained for bacterial culture and sensitivity assessment. We started with removing the screws evident from the field; any buried invisible screws (which could be covered by dense soft tissue or by bone overgrowth) were reached under fluoroscopic guidance.

**Fig. 4:**
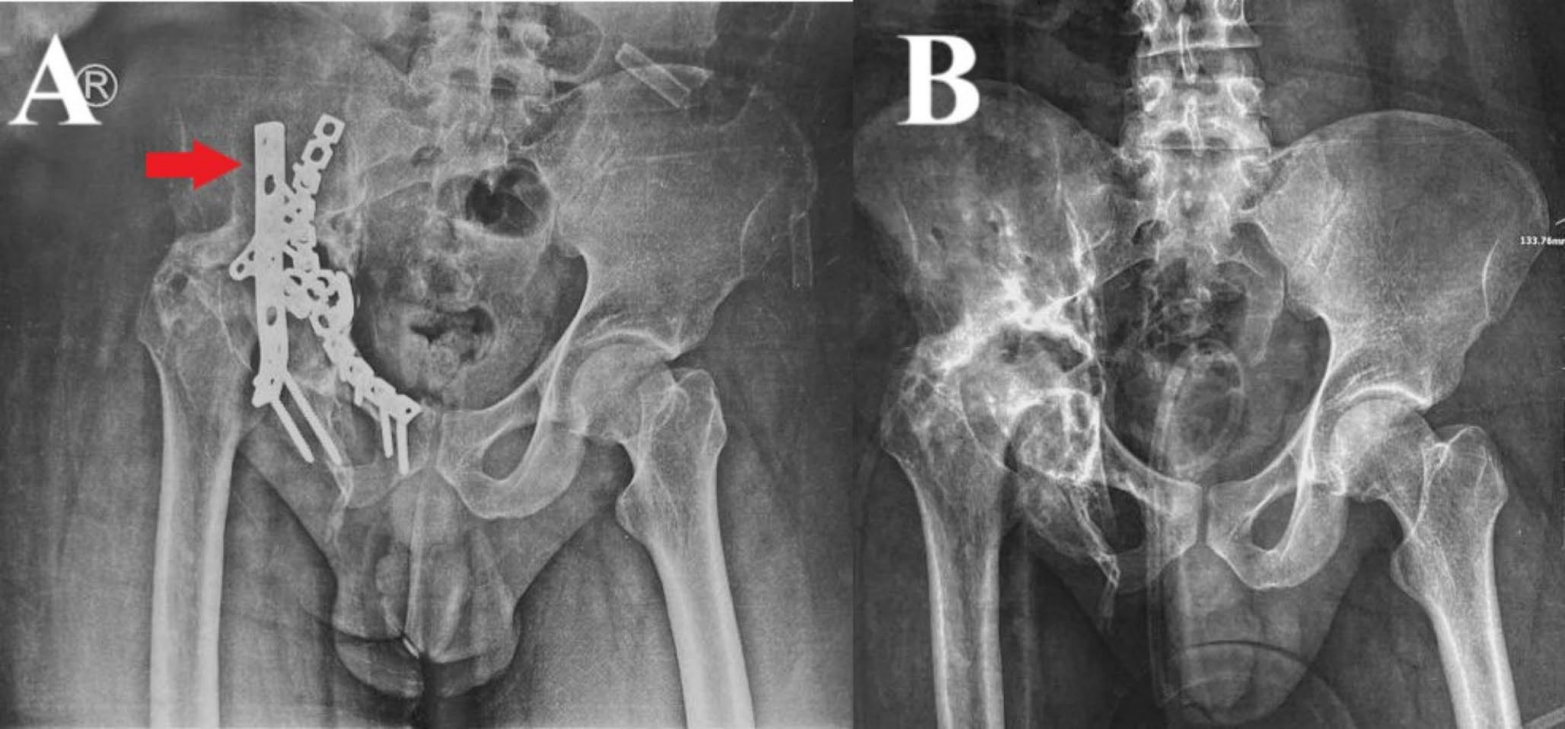
21-year-old male patient who had a T-type associated with a posterior wall fracture, treated by ORIF, hardware was performed through a modified Stoppa approach and a Kocher-Langenbeck approach (to remove the posterior plate indicated by the red arrowhead). **A**, preoperative anteroposterior view. **B**, postoperative. (Patient No. 12 in Supplementary File 2)

In one case (initially managed through a modified Stoppa approach only), an auxiliary lateral (iliac) window of the ilioinguinal approach was opened to avoid the undue over-retraction of the vessels and for better visualization and access of the implant. In cases where an iliac window approach was needed, a Homan retractor was placed medially to protect the L5 nerve root; furthermore, in one case that had a T-type associated with a posterior wall acetabular fracture, a Kocher-Langenbeck approach was required to remove the posterior plate (this was performed on demand of the joint arthroplasty team) (Fig. 4).

The surgical wounds were closed in layers after a negative suction drain was applied to guard against fluid or haematoma collection.

### Postoperative protocol

All patients received antibiotic prophylaxis during their hospital stay and till suture removal. Deep venous thrombosis (DVT) prophylaxis for all patients in the form of low molecular weight heparin during the hospital stay and oral aspirin after discharge. All patients were allowed to bear full weight on the first postoperative day as tolerated. In cases where patients encountered complications (vascular or urinary bladder injuries), instructions from the respective surgical specialties were followed per their management protocols. Follow up at the outpatient clinic after one week for wound assessment and at two weeks for sutures removal.

The patient’s demographic data, operative details, and complication incidents (at any point) were collected (Supplementary File 2). At the last follow-up visits, patients were asked about pain, which was measured using the VAS score, and the patient was asked to describe pain as the following (not present, occasional, or persistent) and their satisfaction after surgery (satisfied, neutral, and dissatisfied).

## Results

Thirteen patients were included, having a mean age of 37.1 ± 14.9 (21 to 65) years, and nine (69.2%) were males. All patients presented with persistent pain, and the underlying indication for hardware removal was a late infection in nine (69.2%) (all of them presented with open sinus) and secondary OA in four (30.8%).

The initial fractures classification per Letournel and Judet classification was T-type fracture in six (46.2%), both columns in five (38.5%), one transverse (7.7%), and one (7.7%) T-type with a posterior wall fracture. All fractures (Seven (53.8%) right and six (46.2%) left) were united at the time of hardware removal, which was performed after the index surgery by a mean of 35.6 ± 20 (11 to 77) months.

The mean operative time was 143.8 ± 36 (100 to 210) minutes, and the mean blood loss was 1573 ± 842 (500 to 3500) CC. Four (30.8%) intraoperative complications were encountered; vascular injury occurred in two (15.4%) patients; one was an external iliac artery injury (treated by direct repair; however, during repair, a spermatic cord injury occurred, which was treated by ligation), and the other was an external iliac vein injury (treated by ligation). One (7.7%) patient had a urinary bladder injury (treated by direct repair and a Foley catheter kept for six weeks). In two (15.4%) patients, the screws were broken and could not be retrieved (Fig. 2D).

The mean hospital stay was 3.2 ± 2.3 (2 to 8) days, and blood transfusion was needed in all patients (a mean of 2.8 ± 1 (7 to 2) units). Postoperative complications were encountered in five (38.5%) patients: three (23.1%) patients (who were operated on through a modified Stoppa approach) had superficial wound infection early postoperatively, which were treated by local debridement and daily dressing, one (7.7%) patient (who had ligation of the external iliac vein) had DVT within the first postoperative week, treated conservatively, and one (7.7%) patient had L5 nerve root injury (presented as weak ankle dorsiflexion) which resolved spontaneously in six months.

After a mean follow up of 11.3 ± 4.4 (6 to 20) months, the VAS score decreased from a preoperative median of 6 (ranging from 2 to 8) to a median score of 1 (ranging from 0 to 6) at the last follow up. 11 (84.6%) patients described the pain as none or occasional, and eight (61.5%) were very satisfied with the results. Persistent pain (15.4%) and dissatisfaction (38.5%) were reported from patients who had secondary OA and were waiting for THA.

## Discussion

Hardware removal after fracture ORIF in the upper or lower limb is controversial; however, in some circumstances, implant removal is considered mandatory [1, 4, 11]. Regarding fracture acetabulum, hardware removal could be performed for peri-implant infection, hardware breakage, hip joint penetration and as a primary step to ease future THA [11, 12, 19–21].

In the current series, hardware removal was successfully performed through anterior approaches for patients who had initial anterior fracture fixation (mainly both column and T-type fractures). Although we had a relatively lower complication rate, which was treated efficiently by members of other surgical specialties, the complications are considered serious, the operative time is long, and there was massive blood loss necessitating blood transfusion in all patients.

**Fig. 5:**
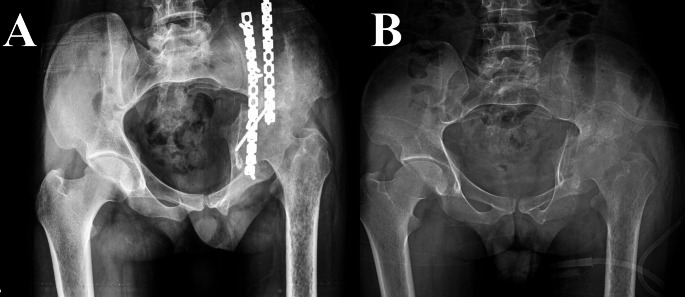
29-year-old male who had a T-type fracture treated by ORIF; the hardware was removed through an Ilioinguinal approach. **A**, preoperative anteroposterior view. **B**, postoperative. (Patient No. 8 in Supplementary File 2)

The main indications for hardware removal in the current series were infection (a clear indication) and the development of secondary OA (Figs. 4 and 5). Hardware removal for secondary OA development might be considered as a relative contraindication; however, in the current series, removal was performed for two main reasons, first is the persistent pain and patient requesting for the procedure, and second is at the request from arthroplasty surgeons, as some of the patients were prepared for THA, and it was requested by the arthroplasty surgeons that the hardware should be removed.

Unfortunately, the literature regarding the exact reasons for hardware removal after acetabular fracture is scarce; however, some studies reported some indications while reporting their experience in managing acetabular fractures.

In a study by Lundin et al., which included 229 patients presented with acetabular fractures and were treated surgically, having a mean age of 60 ± 19 years, 47% were treated through anterior approaches. The authors reported an incidence of reoperation of 21%; most of these cases were due to secondary osteoarthritis (7.4%), followed by infection (3.9%). Furthermore, they reported that in 5.2% (12 patients), the indications for reoperation were surgeon errors, such as misplaced implants, hardware failure, and retained intra-articular fragments. The authors did not comment on the details of hardware removal regarding the approaches and difficulties [22].

In a study aiming to evaluate the reasons for early reoperation after acetabular fractures (defined as surgery within three years after the index operation), Ding et al. evaluated 801 acetabular fractures; 56 (7%) required reoperation to manage infection, 17 of them were operated upon through an anterior approach. Sixty-two (8%) patients underwent early reoperations (nine of them were operated upon through anterior approaches), including conversion to THA (45 patients), ORIF revision (10 patients), and in six patients, hardware removal was required due to joint penetration [19].

In a study by Ordas-Bayonet al. reporting their results after performing THA in patients who had acetabulum fractures, they reported hardware removal only if it came in the way of acetabulum preparation; they reported various approaches for performing such step, either whole implants removal under direct vision (when operating through the posterolateral approach) which could be suitable for hardware implanted through the Kocher-Langenbeck approach, removing hardware through percutaneous incision and under fluoroscopic guidance, or cutting the protruding part of the implants using a diamond burr [12].

In the current series, we described vascular injury at 15.4% and a 7.7% incidence of nerve injury, which was relatively high but was expected due to adhesions and infection. However, vascular and nerve injuries were reported during primary surgeries performed through anterior approaches as well; in a systematic review by Srivastava et al., including ten studies (717 patients) comparing ilioinguinal versus modified Stoppa approach, the incidence of vascular injuries was 5.75% for the ilioinguinal approach and 3.38% for the modified Stoppa approach, furthermore, nerve injuries were reported in 8.56% and 5.54% for the ilioinguinal and modified Stoppa approaches, respectively [14].

Modified Stoppa approach during managing fresh acetabular fractures carries a risk of urinary bladder injury, which could be induced during surgical steps (unattended injury by scalpel or diathermy) or could be injured by vigorous retraction [13, 16]. In cases of hardware removal, the urinary bladder is at a more significant injury risk (which occurred at an incidence of 7.7% in the current series), owing to the presence of adhesions and fragile soft tissues in cases of infection. However, the primary repair under the supervision of urologist surgeons is effective.

***We could provide some recommendations for surgeons willing to remove hardware after acetabular fractures were treated through anterior approaches***:

1. Preoperative patient counseling is paramount to clarifying the indications for surgery, explaining the possible risks, and discussing the expected outcomes.
2. Preoperative evaluation, including a detailed history, complete clinical evaluation, detailed imaging studies (CT if required), and laboratory tests, is vital to determining the definitive indication for hardware removal and performing proper perioperative planning.
3. If vascular injury was suspected at the index surgery, CT angiography could be an essential part of preoperative planning before proceeding with hardware removal.
4. The whole procedure must be performed under direct vision, and fluoroscopy control and guidance are helpful if not possible. Furthermore, the surgeon could use various fluoroscopic views to locate hidden screws [23]. Even more, some authors suggested using intra-operative CT scans for fresh fracture fixation; if surgeons have such a facility, it could be beneficial for locating screws or hardware buried inside the bone [24].
5. To avoid undue important soft tissue structure injuries, excessive and vigorous retraction should be avoided at all costs, as the soft tissues are either tough due to dense fibrous tissue formation or adhesions or could be excessively friable and fragile, such as in cases presented with infection.
6. Various instruments and tools are essential, such as multiple retractors, screw removal sets, and diamond burr, should be part of the operative theater preparation for the surgery.
7. Sometimes, the surgeon might need an auxiliary approach (such as an iliac window in cases treated initially through a Modified Stoppa approach only) to ease manipulation, prevent excessive soft tissue structure retraction, and help with hardware removal and delivery.

The current study’s main limitation is the relatively small number of included patients; however, this should be considered in relation to the paucity of literature on the same subject. Second, the patients’ fate and final functional outcomes were not reported because some patients still needed final management (such as THA).

## Conclusions

Hardware removal from the anterior approaches after acetabular fractures is demanding and carries a high risk for complications, including serious complications, mainly neurovascular bundle injuries. The surgeries should be performed when highly indicated, and the surgical team must be familiar with the anterior approaches; furthermore, the surgical team must include general, vascular, and urological surgeons. Patients indicated for hardware removal for FRI are more satisfied than those presented with secondary OA.

## Electronic supplementary material

Below is the link to the electronic supplementary material.

Supplementary Material 1:CARE checklist for reporting case series

Supplementary Material 2:Included patients’ basic details and outcomes.

## Data Availability

No datasets were generated or analysed during the current study.
