# Supplementary material for: Removing hardware from anterior approaches following acetabular fractures: a challenging yet indicated procedure
Source: Int Orthop. 2024 Nov 27;49(1):249–57. doi: 10.1007/s00264-024-06383-2 (PMC11703930; doi:10.1007/s00264-024-06383-2)
Supplement: Supplementary file 2 — Supplementary Material 2:Included patients’ basic details and outcomes. [file 264_2024_6383_MOESM2_ESM.docx]

| Patient No. | Age | Sex | side | Time from index surgery till hardware removal (months) | Judet-Letournel  Classification of index injury | modified Stoppa  approach | Iliac  window | Pararectus  approach | Ilioinguinal  approach | Indication  (Diagnosis) | Follow up  (months) | Pain description (VAS score) | | Satisfaction reported at last follow up |
| --- | --- | --- | --- | --- | --- | --- | --- | --- | --- | --- | --- | --- | --- | --- |
|  |  |  |  |  |  |  |  |  |  |  |  | Preoperative | At last follow up |  |
| 1 | 44 | M | LT | 19 | both column | YES | YES | No | No | Infection | 6 | Persistent (7) | No (0) | satisfied |
| 2 | 65 | M | RT | 26 | T | No | No | YES | No | infection | 10 | Persistent (7) | No (0) | satisfied |
| 3 | 23 | M | RT | 30 | T | YES | YES | No | No | Infection | 12 | Persistent (8) | No (0) | satisfied |
| 4 | 36 | M | RT | 36 | T | YES | YES | No | No | Infection | 8 | Occasional (5) | Occasional (2) | neutral |
| 5 | 30 | F | LT | 51 | both column | YES | YES | No | No | OA | 16 | Persistent (6) | Persistent (6) | dissatisfied |
| 6 | 34 | F | RT | 16 | both column | YES | YES | No | No | Infection | 10 | Occasional (4) | No (0) | satisfied |
| 7 | 32 | F | RT | 63 | T | YES | YES | No | No | Infection | 8 | Occasional (5) | Occasional (1) | neutral |
| 8 | 29 | M | LT | 11 | T | No | No | No | YES | OA | 16 | Persistent (7) | Occasional (2) | satisfied |
| 9 | 60 | M | LT | 42 | both column | YES | YES | No | No | OA | 6 | Persistent (6) | Occasional (3) | satisfied |
| 10 | 58 | M | LT | 20 | both column | YES | YES | No | No | OA | 20 | Persistent (8) | Persistent (6) | dissatisfied |
| 11 | 23 | F | RT | 77 | Transverse | YES | No | No | No | Infection | 16 | Persistent (6) | Occasional (2) | dissatisfied |
| 12 | 21 | M | RT | 48 | T+PW | YES | No | No | No | Infection | 9 | Occasional (3) | No (0) | satisfied |
| 13 | 27 | M | LT | 24 | T | YES | YES | No | No | infection | 10 | Occasional (2) | No (0) | satisfied |

| Patient No. | Hospital stay (Days) | Operative time  (Minutes) | Blood Loss  (cc) | Blood Transfusion  (units) | Complications | | | | | |
| --- | --- | --- | --- | --- | --- | --- | --- | --- | --- | --- |
|  |  |  |  |  | nerve injury | Vascular Injury | DVT | Infection | Bladder injury | Others |
| 1 | 8 | 210 | 3500 | 7 | No | External iliac artery | No | Yes (superficial) | No | spermatic cord ligation |
| 2 | 2 | 150 | 2000 | 3 | No | No | No | No | No |  |
| 3 | 5 | 140 | 2500 | 3 | No | External iliac vein | YES | No | No | screw broken inside the bone |
| 4 | 3 | 120 | 1700 | 3 | No | No | No | Yes (superficial) | No |  |
| 5 | 2 | 160 | 1600 | 2 | No | No | No | Yes (superficial) | No |  |
| 6 | 3 | 180 | 1100 | 2 | L5 Nerve root | No | No | No | No |  |
| 7 | 2 | 200 | 2400 | 3 | No | No | No | No | YES |  |
| 8 | 4 | 120 | 1200 | 2 | No | No | No | No | No |  |
| 9 | 3 | 130 | 500 | 2 | No | No | No | No | No |  |
| 10 | 2 | 155 | 1300 | 3 | No | No | No | No | No |  |
| 11 | 2 | 200 | 900 | 2 | No | No | No | No | No | screw broken inside the bone |
| 12 | 2 | 100 | 1000 | 2 | No | No | No | No | No | MR to posterior plating |
| 13 | 3 | 110 | 750 | 2 | No | No | No | No | No |  |
